# Spatial Transferability of Habitat Suitability Models of *Nephrops norvegicus* among Fished Areas in the Northeast Atlantic: Sufficiently Stable for Marine Resource Conservation?

**DOI:** 10.1371/journal.pone.0117006

**Published:** 2015-02-13

**Authors:** Valentina Lauria, Anne Marie Power, Colm Lordan, Adrian Weetman, Mark P. Johnson

**Affiliations:** 1 Ryan Institute, School of Natural Sciences, National University of Ireland, Galway, University Road, Galway, Ireland; 2 Department of Zoology, School of Natural Sciences, Ryan Institute, National University of Ireland, Galway, University Road, Galway, Ireland; 3 Marine Institute, Rinville, Oranmore, Co. Galway, Ireland; 4 Marine Scotland Science, Marine Laboratory, Aberdeen, Scotland; McGill University, CANADA

## Abstract

Knowledge of the spatial distribution and habitat associations of species in relation to the environment is essential for their management and conservation. Habitat suitability models are useful in quantifying species-environment relationships and predicting species distribution patterns. Little is known, however, about the stability and performance of habitat suitability models when projected into new areas (spatial transferability) and how this can inform resource management. The aims of this study were to model habitat suitability of Norway lobster (*Nephrops norvegicus*) in five fished areas of the Northeast Atlantic (Aran ground, Irish Sea, Celtic Sea, Scotland Inshore and Fladen ground), and to test for spatial transferability of habitat models among multiple regions. *Nephrops* burrow density was modelled using generalised additive models (GAMs) with predictors selected from four environmental variables (depth, slope, sediment and rugosity). Models were evaluated and tested for spatial transferability among areas. The optimum models (lowest AICc) for different areas always included depth and sediment as predictors. Burrow densities were generally greater at depth and in finer sediments, but relationships for individual areas were sometimes more complex. Aside from an inclusion of depth and sediment, the optimum models differed between fished areas. When it came to tests of spatial transferability, however, most of the models were able to predict *Nephrops* density in other areas. Furthermore, transferability was not dependent on use of the optimum models since competing models were also able to achieve a similar level of transferability to new areas. A degree of decoupling between model ‘fitting’ performance and spatial transferability supports the use of simpler models when extrapolating habitat suitability maps to different areas. Differences in the form and performance of models from different areas may supply further information on the processes shaping species’ distributions. Spatial transferability of habitat models can be used to support fishery management when the information is scarce but caution needs to be applied when making inference and a multi-area transferability analysis is preferable to bilateral comparisons between areas.

## Introduction

Species distribution models (SDMs), also called habitat models, habitat preference, habitat suitability or habitat distribution models, are empirically-defined models relating field observations (e.g. presence-only, presence-absence or abundance only) to environmental variables, with the aim of quantifying species-environment relationships and predicting species occurrence and/or density at unsurveyed locations [1,2]. The application of such models has become an important tool to address issues in ecology, biogeography, conservation planning and more recently in climate change research [3–5]. As well as improving knowledge about how environmental changes might affect species’ geographical distributions [6,7], SDMs represent a useful tool to inform management decisions. One important application of SDMs is in the area of fisheries management; for example, identifying nursery areas of commercially important fish species [8], spatial distributions of vulnerable species, such as elasmobranchs [4] or predicting the distribution patterns of commercially exploited species in response to future climate change scenarios [5].

A number of statistical techniques have been developed to model the habitat of species (reviewed in [7]) such as Generalised Additive Models (GAMs), neural networks, and boosted decision trees [6,9]. In general, applications of SDMs are limited to one region by splitting the observation data into two datasets named “training” and “testing”. The model is fitted on the training data and then its performance is evaluated on the testing data [2,6].

Although this type of validation is widely used in SDM it has some limitations that can affect model performance: local cross-validation cannot assess model generalizability, also termed ‘transferability’ which refers to a model’s capacity to predict species’ distribution when transferred into another geographical region or time period [10–13]. Consequently testing for model transferability has been recommended to complement standard procedures of model evaluation [14–18]. Generally a model is assumed to be perfectly transferable when it captures species-environment relationships and these do not vary across contexts [19]. Nevertheless, some variability may occur in model behaviour between regions due to the differences in explanatory variables (i.e. range of values; [19]).

Although the number of studies on transferability of SDMs has increased in recent years [13,20–23], this particular aspect of habitat modelling is still being developed and subject to debate [24]. Typically studies on transferability of SDMs are limited to two regions (Table 1; but see [25,26] and very little is known about the stability and performance of a model when transferred to multiple areas. Spatial transferability of habitat models may have particular relevance in the context of conservation of marine systems and can be used to support fisheries management policies. Only a limited number of studies have examined the spatial transferability of SDMs in marine systems (Table 1). A greater understanding of the confidence in applying SDMs would support resources management when the information on a specific marine area is scarce, which is often the case.

**Table 1:** Case studies where spatial transferability has been tested in habitat suitability models.

System	Study area	Application	N of regions/sites compared	Model used	Reference
Terrestrial	Switzerland	Amphibian	5	GLMs	[26]
Terrestrial	North America	Bird	4	MAXENT and GARP	[25]
Terrestrial	Switzerland	Birds	4	GLMs	[23]
Fresh waters	Finland	Fish	4	Habitat suitability curves	[56]
Terrestrial	North America, Europe & worldwide	True bug	3	MAXENT	[57]
Terrestrial	Belgium	Butterflies	3	GLMs	[58]
Terrestrial	Switzerland & Austria	Plants	2	GLMs; GAMs	[13]
Terrestrial	Germany	Insects	2	GLMs	[17]
Marine	Mediterranean and Australia	Seaweed	2	MAXENT	[24]
Fresh waters	Quebec	Fish	2	NHM (hydrodynamic & biological models)	[59]
Marine	Baltic Sea	Fish	2	GAMs	[60]
Fresh waters	Virginia	Benthic fish	2	Multiple logistic regression	[61]
Fresh waters	New England	Fish	2	Habitat suitability criteria (HSC)	[62]

Here we describe a study where SDMs were created for five different fished areas in the Northeast Atlantic with an evaluation of model transferability. The distribution of the Norway lobster (*Nephrops norvegicus;* hereafter referred to as *Nephrops*) was modelled as a function of environmental predictors: depth, sediment type, slope and rugosity.

*Nephrops* supports one of the most valuable fisheries from the Northeast Atlantic to the Mediterranean [26–28]. Although *Nephrops* landings have generally increased over the past five decades reaching 66,554 tonnes in 2010 in Europe, some latitudinal differences exist, with some regions (British Isles) being more productive than others (Portugal, Bay of Biscay) possibly as a result of fisheries impacts on stocks [29]. *Nephrops* live in shallow (20–30 cm) burrows in soft stable mud at depths ranging from 20 to 800 m [30,31]. Many discrete stocks exist in the Northeast Atlantic and their boundaries often reflect presence of large-scale mud patches [32]. The presence of suitable sediment is considered a key factor for *Nephrops* habitat selection and distribution, however the relationship between *Nephrops* burrow density and sediments appears to be non-linear and stock-specific [32,33]. A limited number of studies have investigated the relationship between *Nephrops* abundance and sediment characteristics or broad scale environmental parameters in the Northeast Atlantic [33,34], but information specifying the context-specific versus general habitat requirements that determine *Nephrops* habitat selection is lacking.

The importance of mapping species distributions for protection of habitats and resource management has been highlighted in the European Marine Strategy Framework Directive [35] and Common Fisheries Policy (2013)[36]. This study characterises the most important environmental factors associated with the key distribution areas of *Nephrops* in the Northeast Atlantic while establishing the extent of a generally applicable definition for *Nephrops* habitat based on benthic variables.

## Material and Methods

### Fished areas for *Nephrops*

**Fig 1:**
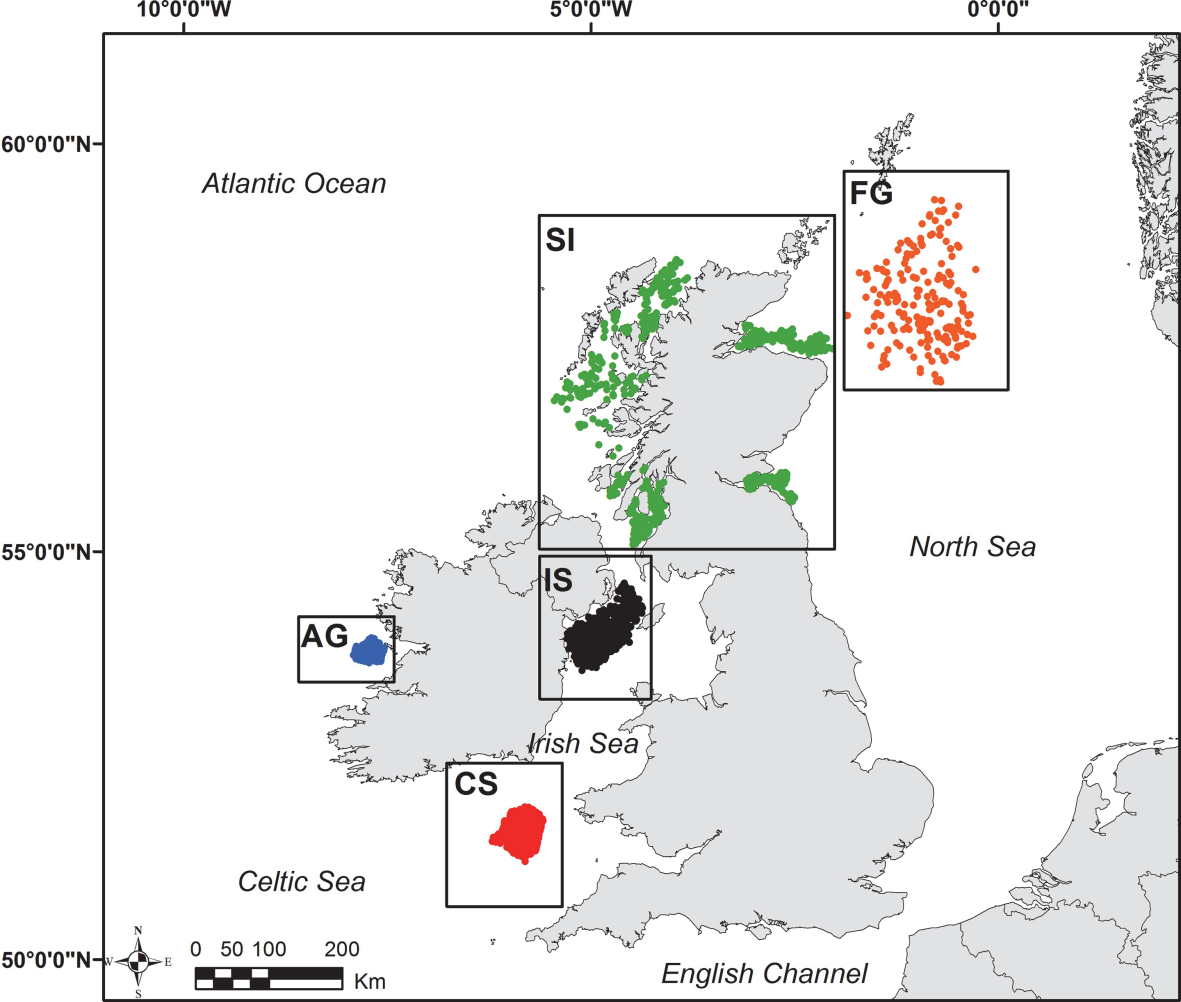
Study area and video tow locations for *Nephrops* fished areas in the Northeast Atlantic. The sampling stations for are indicated as follows: (AG) Aran ground (in blue); (CS) Celtic Sea (in red); (IS) Irish Sea (in black); (SI) Scotland inshore waters (in green) and (FG) Fladen ground (in orange).

Our study areas reflect important fishing grounds on the Northeast Atlantic continental shelf (Fig. 1). The five fished areas are based on Functional Units (FUs) used by the International Council for the Exploration of the Sea (ICES) Working Group on Nephrops Stocks for management and reporting [27]. The fished areas considered in this study are: the Aran ground (52°51'-53°8'N; 9°46'-10°21'W), Celtic Sea (50°56'-51°39'N; 5°47'-6°49'W), Irish Sea (53°23'- 54°34'N; 4°45'-6°12'W) and Fladen ground (57°30'- 59°54'N; 1°33'-1°38'W). Scottish inshore grounds (55°11'-58°39'N; 1°38'-7°40'W) represent an amalgamation of several smaller FUs (e.g. Firth of Forth, Moray Firth, North Minch, South Minch and Clyde). European *Nephrops* fisheries have increased significantly over time and recent landing statistics show that nearly 70% of the catch is fished by UK and Irish fleets [29].

### *Nephrops* data

*Nephrops* burrow density was estimated by underwater TV (UWTV) surveys carried out in UK and Irish waters between 2002–2011 where a sledge-mounted TV system was towed over the sea bed for 10 minutes travelling approximately 200 m at 0.8 knots [33,37]. For the UWTV surveys no specific permissions were required for these locations/activities, in addition the field studies did not involve endangered or protected species. The numbers of *Nephrops* burrows were counted and these data were used to estimate a mean adjusted density (number of burrows m^-2^) for each stock. Burrows are a proxy for the number of individuals as there is approximately one adult per burrow. Adjustments are made to burrow counts for stock assessment purposes, for example taking into account edge effects (see [38]). *Nephrops* burrow densities among the fished areas ranged from a minimum value of 0.34 m^-2^ in the Fladen ground to a maximum of 1 m^-2^ in the Irish Sea (Table 2). Although some natural year-to-year variation can occur in *Nephrops* densities, these did not show any significant trend over time in the different areas (Table 2). The densities were consequently treated as a steady state pattern in each area over the study period.

**Table 2:** Summary of *Nephrops* data and temporal trend for all fished areas.

Fished area	N of data points	Mean *Nephrops* density (m^-2^)	Time period	Slope(±SE)	p value	R^2^%	CV
Aran ground (AG)	78	0.66	2002–2009	-0.02(±0.03)	>0.05	-0.06	0.40
Celtic Sea (CS)	201	0.54	2006–2008; 2011	-0.06(±0.02)	>0.05	0.69	0.95
Irish Sea (IS)	563	1.00	2003–2011	-0.01(±0.01)	>0.05	-0.11	0.58
Scotland Inshore (SI)	601	0.66	2008–2011	-0.00(±0.01)	>0.05	-0.99	0.69
Fladen ground (FG)	196	0.34	2008–2010	-0.07(±0.00)	>0.05	0.97	0.85

*Nephrops* density is expressed as the mean adjusted burrow density m^-2^; R^2^: adjusted coefficient; CV: Coefficient of Variation.

### Environmental predictors

**Fig 2:**
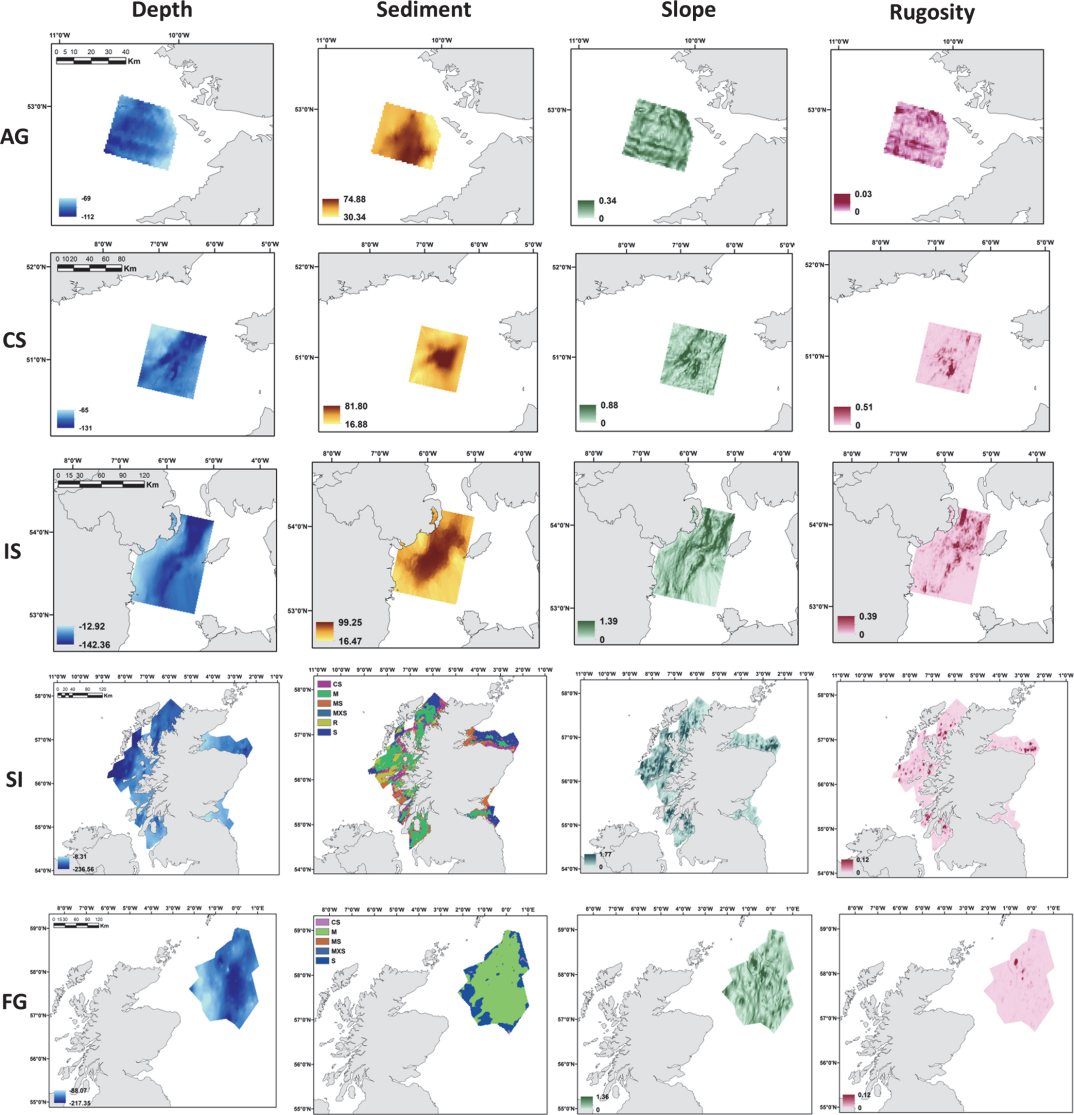
The spatial patterns of the environmental variables used to map the habitat suitability models for each fished area including: depth (m), seabed sediment type (percentage of silt plus clay) for Aran Grounds (AG), Celtic Sea (CS) and Irish Sea (IS); sediment classes (fine mud (M), muddy sand (MS), sand (S), coarse sand (CS), mixed sediments (MXS) and rocks (R) for Scottish Inshore (SI) and Fladen ground (FG) (see the main text for details). Slope (degrees) the maximum possible range is from 0 to 90 degrees, with low values corresponding to flat ocean bottom (or areas of sediment deposition) while higher values indicate potential rocky ledges. Terrain ruggedness or rugosity values range from 0 (no terrain variation) to 1 (complete terrain variation).

For habitat modelling, depth, slope, terrain ruggedness (or rugosity) and sediments were used as predictors of *Nephrops* densities (Fig. 2). ArcGIS’s implementation of the Lambert Azimuthal Equal Area projection (ETRS 1989) was chosen as appropriate for use within the regional extent of our study. This is an equal area map projection and designed to minimise area distortions at mid-latitudes with east-west orientation. Digital continuous maps of depth were derived from a reprojection of the British Oceanographic Data Centre (BODC) GEBCO_08 data; available at http://www.gebco.net. GEBCO is world bathymetry dataset with 30 arc seconds x 30 arc seconds resolution. Extracted raster size for estimation of benthic variables was 879 x 879 m.

Quantitative descriptors of the seabed terrain such as slope and rugosity were derived from the depth continuous map using the Benthic Terrain Modeller tool in ArcGIS 10.1 [39]. Slope (expressed in degrees) describes the rate of change in elevation over distance; the maximum possible range is from 0 to 90 degrees, with low values corresponding to flat terrain and higher values to steeper terrain (Fig. 2). In the marine environment, low values of slope correspond to a flat ocean bottom (or areas of sediment deposition) while higher values indicate potential rocky ledges.

Terrain rugosity is an indicator of soft/hard-bottom habitat. It captures variability in slope and aspect into a single measure and gives an indication of the bumpiness and complexity of the seafloor. This parameter is unit less, with rugosity values ranging from 0 (no terrain variation) to 1 (complete terrain variation) (Fig. 2). Generally, soft seabed substrate corresponds to low terrain rugosity while high terrain rugosity potentially indicates rocky seabed.

Sediment data (expressed as the percentage of silt plus clay) for the Aran ground, Celtic Sea and Irish Sea (Fig. 2) were collected using a Day grab and particle size analysis was carried out using a Low Angle Laser Light Scattering method. Locations for each video tow (burrow density estimate) were matched to the nearest sediment grab location using a spatial join in ArcGIS. Continuous digital sediment maps were created by interpolation (kriging) of the variogram of the % of silt and clay in ArcGIS. Different smoothing functions were tested for each area, and the best one was selected on the basis of individual plots and variogram model fits. As the information on sediment was not consistent for the Scottish Inshore and Fladen grounds, seabed sediment information was based on the UKSeaMap predicted seabed habitat map (available at http://jncc.defra.gov.uk/page-2117). Sediment classes (based on EUNIS habitat classification level 4) were aggregated into six broad classes: fine mud (M), muddy sand (MS), sand (S), coarse sand (CS), mixed sediments (MXS) and rocks (R) (Fig. 2). This information was extracted using ArcGIS at each survey location for all the Scottish areas.

### Statistical analysis and mapping

**Fig 3:**
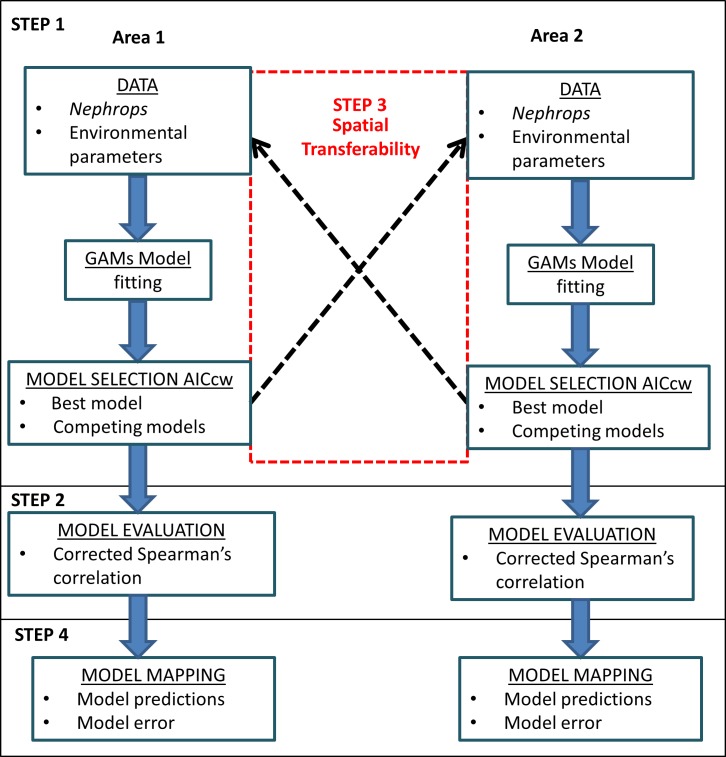
Simplified design of model construction, evaluation and analysis of transferability between two areas.

A simplified analytical design is presented in Fig. 3 which shows the process for assessing SDMs in two areas, including an evaluation of transferability. The process includes four main steps: (1) model selection, (2) model evaluation, (3) model transferability between areas, and (4) model mapping.

1. **Model selection**. GAMs are nonparametric regression techniques that allow for the modelling of relationships between variables without specifying any particular form for the underlying regression function. The use of smooth functions to relate predictor variables to the dependent variable gives GAMs greater flexibility over linear (or other parametric) types of models. In addition, the use of GAMs has been shown to be potentially more robust than others (such as GLMs) when transferred from one geographical region to another [16].

The full model included four environmental predictors:

Nephropsdensity~s(sediment)+s(depth)+s(slope)+s(rugosity)

Starting from the full model, the most parsimonious model (or best model) was selected on the basis of the lowest Akaike Information Criterion (AIC), corrected for small sample size (AICc). This approach selects the model with the best balance between overfitting and precision and avoids problems of multiple testing among explanatory variables [40]. A set of candidate models was compared using difference in AICc between the top-ranked and current model (delta AICc), and by calculating the AICc weight (the scaled likelihood that each model is the best description of the data [40]. Competing models were selected when their AICc was within 2 of the minimum [40]. All modelling was carried out using the R software [41] and its mgcv library [42].

Residuals from fitted models were examined for overdispersion and plots of residuals were examined to check for normality, homogeneity and independence [42,43]. As the video surveys concentrate on known grounds, zero density estimates are relatively rare and residuals generally supported the assumption of normally distributed errors. In one case, (Scotland Inshore) a log normal link may have been more appropriate, but this would have prevented direct transferability to other regions, so all models were fitted with the assumption of normally distributed errors. Differences in error distribution were assumed to be one of the potential factors contributing to limits on the effectiveness of transferability. The predictor variables were not collinear, with variance inflation factors less than 2 [40].

1. **Model evaluation**. Model goodness of fit was compared using the deviance and coefficient of determination (Adj-R^2^). Models were also evaluated internally by comparing predictions in relation to the observation with Spearman's rank correlation test (r_s_) corrected for spatial autocorrelation and implemented in SAM software [44,45].
2. **Model transferability**. To assess model transferability in space we used the habitat model developed for one specific fished area with the predictor variables from other areas (for example the model developed for Aran ground was fitted to the Celtic Sea and Irish Sea data). Spearman's rank correlation test corrected for spatial autocorrelation was used to compare new predictions and observations [4,8]. Model behaviours were also compared qualitatively by checking if the same variables were important across regions [17] and comparing the functional form of the relationships [11]. In order to test for consistency of model transferability, competing models were also transferred to other areas when applicable. Because of the different sediment proxies used for model construction, it was not possible to test for spatial transferability among all five areas, therefore models that used sediment grabs data (%silt plus clay) (e.g. Aran ground, Celtic Sea and Irish Sea) were cross-transferred, while models that used EUNIS sediment data (i.e. Fladen ground and Scottish Inshore) were reciprocally transferred.

Models were considered fully transferable (i.e. equality of parameters between estimation and application contexts) if they met the following two conditions: 1) the internal evaluation of models fitted in two different areas were similar ([13]); and 2) the transfer of a model fitted in one area to another area passed the Spearman’s correlation test corrected for spatial autocorrelation (positive correlation between observed and predicted values in the new area).

1. **Model mapping**. Maps of *Nephrops* predictions were constructed within the raster and rgdal libraries in R [46,47] and then visualised in ArcGIS. The model error (defined as the absolute difference between observed and predicted species abundance) was also used to check and illustrate model fit. The spatial distribution of the model error was mapped by kriging for each area, scaled between 0 to 1, with a value of 1 corresponding to the maximum possible prediction error [8]. Model errors were generally spatially autocorrelated, as judged from semivariograms. There is an ongoing debate in the spatial distribution modelling literature about how to account for or remove spatial autocorrelation from inference. As we are primarily interested in transferability, we chose not to explicitly model spatial dependence as transferability is itself a test of whether spatial dependence in the area used for model fitting is important to the effectiveness of predictions in a new area (with new spatial dependencies).

## Results

### Environmental factors relevant to *Nephrops* habitat

**Fig 4:**
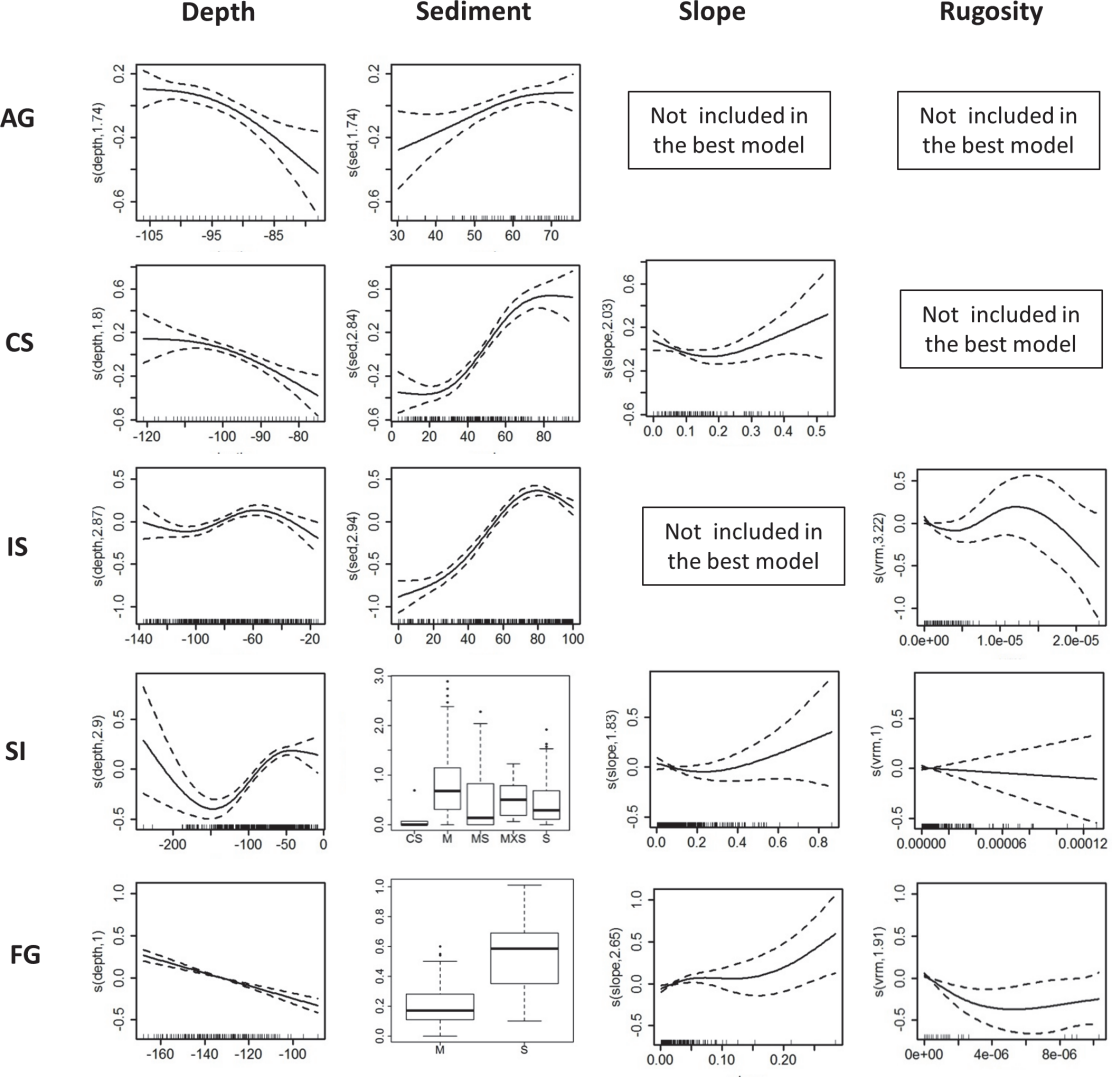
Partial GAM plots for the best Gaussian models for each fished area. Each plot represents the variable’s response shape, independent of the other variables, in relation to the predicted *Nephrops* burrow density in the model. Slope (expressed in degrees) describes the rate of change in elevation over distance, the maximum possible range is from 0 (flat terrain) to 90 (steeper terrain) degrees; vrm: terrain rugosity (captures variability in slope and aspect into a single measure, it ranges from 0 (no terrain variation) to 1 (complete terrain variation). The ranges of environmental variables are represented on the x-axis and the related change in *Nephrops* density is represented on the y-axis (logit scale). The degree of smoothing is indicated in the y-axis label. The dotted lines represent the 95% confidence intervals around the response curve. For the Scotland Inshore and Fladen ground models the effects of each sediment class are shown as a boxplot. Sediments are divided into 6 classes (based on EUNIS classification level 4): Fine mud (M), muddy sand (MS), sand (S), coarse sand (CS), mixed sediments (MXS) and rocks (R). For more details see the main text.

Depth and sediment type were predictors of the optimum *Nephrops* habitat models in all areas (Table 3; Fig. 4). Rugosity was found to be a predictor of *Nephrops* habitat suitability in the Irish Sea, Scotland inshore waters and Fladen ground models. Slope was included in the best model for the Celtic Sea, Scotland inshore waters and Fladen ground (Table 3; Fig. 4). The optimum models were generally good predictors of variation in burrow density, with over 40% of deviance explained, except for the Scotland Inshore—although the model was still able to provide an adequate explanation of spatial variation in densities (r_s_ value).

**Table 3:** Selected models for *Nephrops* habitat suitability.

Fished area	Model selection							Model evaluation	
	depth	Sed (%silt plus clay)	slope	rugosity	AICw	Adj-R^2^	Dev %	r_s_	p value
Aran ground (AG)	X	X			0.42	0.50	53.0	0.52	***
Celtic Sea (CS)	X	X	X		0.45	0.56	57.9	0.73	***
Irish Sea (IS)	X	X		X	0.69	0.41	41.9	0.59	***
	**depth**	**Sed (EUNIS)**	**slope**	**rugosity**	**AICw**	**Adj-R^2^**	**Dev %**	**r_s_**	**p value**
Scotland Inshore (SI)	X	X	X	X	0.28	0.20	21.6	0.32	*
Fladen ground (FG)	X	X	X	X	0.98	0.39	41.9	0.67	**

Predictors include depth, sediment (sed), slope and rugosity (variables included in model are marked with X). Only the best supported models are shown; AICw: Akaike’s Information Criteria (corrected for small sample size) weights, values range from 0 to 1, and high values indicate strong support for the associated model. Goodness of fit is summarized by Dev%: percentage of deviance explained; and Adj-R^2^: adjusted coefficient of determination. Sed: sediment describing % silt plus clay for the Aran ground, Celtic Sea and Irish Sea. Sed (EUNIS): sediment based on EUNIS classification level 4 (see text for more details) including only Mud (M) for the Scotland Inshore and Fladen ground models. Significance value of Spearman's rank correlation test (r_s_) (corrected for spatial autocorrelation) between observed and predicted densities (r_s_) labelled as ***: p<0.001, **: p<0.01, *: p<0.05.

The influences of environmental predictors are shown in Fig. 4. Depth had nonlinear effects on densities in all fished areas except the Fladen ground. Three fished areas had higher *Nephrops* densities in deeper waters, while the Irish Sea and Scotland Inshore also predicted peak densities at shallower depths. The shape of the smoother of sediment (% of silt plus clay) for the Aran grounds, Celtic Sea and Irish Sea indicated a positive nonlinear relationship associated with higher content of silt and clay (60–80%). Slope had a positive nonlinear relationship with *Nephrops* densities in the Celtic Sea, Scottish inshore waters and Fladen ground with an inflexion at circa 0.2 (Fig. 4). The shape of the smoother for rugosity suggests that *Nephrops* densities are lower in areas of rougher seabed, but there was variation between areas in the form of the relationship across the range of rugosities (Fig. 4).

A comparison among competing models for fished areas indicated clear evidence for the model with the lowest AICc in three of the five fished areas (Table 4, there was no competing model for the Fladen ground due to the strong support for the optimum model; AICw 0.98, Table 3). The evidence ratios (between AICw of best and competing models) for the Irish Sea and Aran Grounds exceed 2.8, implying that the optimum model in each case was nearly 3 times more likely than the competing model. In contrast, the optimum Celtic Sea model was 1.8 times as probable as the competing model, while for Scotland Inshore, the optimum model was not clearly better than the best performing alternatives.

**Table 4:** Competing habitat suitability models for *Nephrops* in fished areas.

Fished area	Model selection								Model evaluation	
		depth	Sed (%silt plus clay)	slope	rugosity	AICw	Adj-R^2^	Dev %	r_s_	p value
Aran ground (AG)	M1	X	X		X	0.15	0.49	52.9	0.53	***
Celtic Sea (CS)	M1	X	X	X	X	0.24	0.56	58	0.74	***
	M2	X	X			0.18	0.55	56.5	0.72	***
Irish Sea (IS)	M1	X	X	X	X	0.24	0.41	41.9	0.59	***
		**depth**	**Sed (EUNIS)**	**slope**	**rugosity**	**AICw**	**Adj-R^2^**	**Dev %**	**r_s_**	**p value**
Scotland Inshore (SI)	M1	X	X	X		0.28	0.20	21.6	0.45	*
	M2	X	X		X	0.21	0.19	20.9	0.45	*
	M3	X	X			0.15	0.19	20.9	0.44	*

There was no competing model for the Fladen ground due to the strong evidence (AICw) for the optimum model. See legend Table 3 for more details and explanation of terms.

### Predicting the spatial distribution of *Nephrops* and its uncertainty

**Fig 5:**
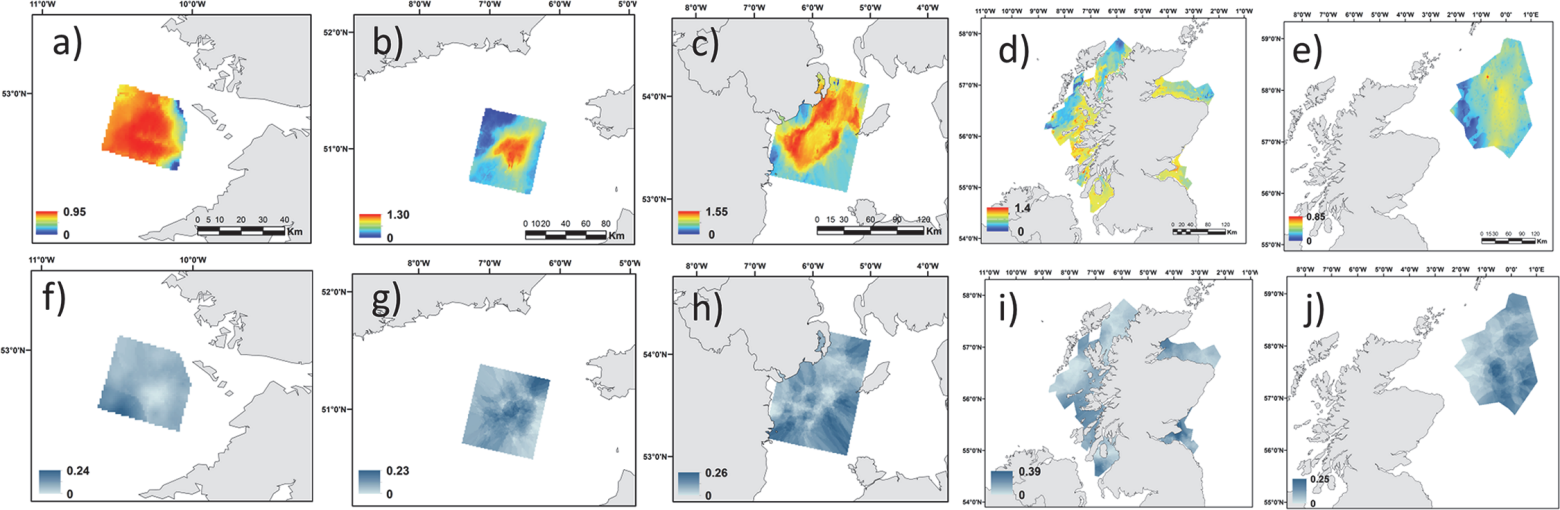
Mapped *Nephrops* prediction probabilities and associated error (below) for the fished areas: (a) Aran ground, (b) Celtic Sea, (c) Irish Sea, (d) Scotland inshore waters and (e) Fladen ground (f). Prediction error: 0 and 1 correspond to the minimum and maximum possible errors, respectively.

Maps of predicted *Nephrops* burrow densities for all areas (Fig. 5) emphasize the associations with high percentages of silt and clay in the Aran grounds, Celtic Sea and Irish Sea (see also Fig. 2). The results reflect the collection of video tow data over areas with peak predicted densities towards the centre of the survey point locations.

Prediction errors associated with each habitat map showed that, for the Aran grounds and Irish Sea, the prediction uncertainty was lower in the central area (Fig. 5F and Fig. 5H). Conversely the error tended to be higher in the central part of the Celtic Sea (Fig. 5G). The error map for the Scottish inshore waters revealed that higher model uncertainty corresponded to areas with higher predictions (Fig. 5D and Fig. 5I) while in the Fladen, the error was higher in the southeast and central parts of the patch (Fig. 5J).

### Model transferability between fished areas

Positive correlations (Spearman’s correlation test corrected for spatial autocorrelation) were found between observed and predicted densities in six cases when optimum models from one area were tested in another area (Table 5). In these cases, the models were transferable as they met the criteria for an adequate model in the training data set and predictive capacity in the test cases. The Scottish inshore waters and Fladen ground models could not be reciprocally transferred and the correlations using the optimum model for one area in the other were even negative (r_s_ = -0.18 and r_s_ = -0.11; Table 4).

**Table 5:** Model transferability between different fished areas.

	Transferability				
Fished area fitted	Area transferred to	r_s_	p value	Similarity between fished areas	Score
Aran ground	Celtic Sea	0.75	***	yes	2/2
	Irish Sea	0.19	*	yes	2/2
Celtic Sea	Aran ground	0.52	***	yes	2/2
	Irish Sea	0.29	*	yes	2/2
Irish Sea	Aran ground	0.41	*	yes	2/2
	Celtic Sea	0.63	***	yes	2/2
Scottish Inshore	Fladen ground	-0.18	>0.05	yes	1/2
Fladen ground	Scottish Inshore	-0.11	>0.05	yes	1/2

Significance Spearman's rank correlation test (corrected for spatial autocorrelation) (r_s_) given as ***: p<0.001, **: p<0.01, *: p<0.05. Transferability between fished areas (2/2 criteria) implies that the internal evaluations of models were similar ([13]; see main text for more details) and that predictions from a model to a new area where it is evaluated are positively correlated with observations.

Where they were transferable, the competing models (presented in Table 4) performed just as well as the models with the lowest AICc, with very similar predicted to observed correlations (Table 6). The alternative Scotland inshore models did not transfer well to the Fladen grounds, although this was no different to the performance of the optimum inshore model. Akaike weights were relatively high compared to other fits for the Aran ground and Irish Sea and this was reflected in good transferability to the other regions, however, models with a lower weight performed just as well in terms of transferability.

**Table 6:** Transferability of competing models among different fished areas.

	Competing model						Transferability			
Area where model parameterised		depth	Sed (%silt plus clay)	Slope	rugosity	Area where transferred	r_s_	p value	Similarity between fished areas	Score
Aran ground	M1	X	X		X	Celtic Sea	0.74	***	yes	2/2
	M1	X	X		X	Irish Sea	0.18	*	yes	2/2
Celtic Sea	M1	X	X	X	X	Aran ground	0.52	***	yes	2/2
	M2	X	X				0.50	***	yes	2/2
	M1	X	X	X	X	Irish Sea	0.30	*	yes	2/2
	M2	X	X				0.30	*	yes	2/2
Irish Sea	M1	X	X	X	X	Aran ground	0.42	*	yes	2/2
	M1	X	X	X	X	Celtic Sea	0.63	***	yes	2/2
**Area where model parameterised**		**depth**	**Sed (EUNIS)**	**Slope**	**rugosity**	**Area where transferred**	**r_s_**	**p value**	**Similarity between fished areas**	**Score**
Scotland Inshore	M1	X	X	X		Fladen ground	-0.13	>0.05	yes	1/2
	M2	X	X		X		-0.16	>0.05	yes	1/2
	M3	X	X				-0.17	>0.05	yes	1/2

Transferability reflects good internal evaluation and a positive correlation between the observed and predicted values after a model is transferred to a novel area. Predictors include depth, sediment (sed), slope and rugosity (variables included in model are marked with X). See legend Table 5 for more details.

## Discussion

### Predicting *Nephrops* habitat suitability

Benthic variables were robust predictors of *Nephrops norvegicus* burrow densities. Depth and sediment type were included as predictors for *Nephrops* potential habitat in all five fished areas, while rugosity and slope were found to be important in only some cases (Table 3). Generally *Nephrops* can be found at depths between 10 and 800 m [30,34], however our results suggest that the relationship between density and depth varies between areas and can be complex (Fig. 4). We believe that a key environmental factor driving *Nephrops* distribution patterns is the availability of suitable sediments for burrow building and the availability of such sediment may have local dependencies on hydrography, geology and bathymetry that can result in the observed variations among areas.

Previous studies [33,34] of the relationship between *Nephrops* burrow density and sediment composition in Scotland (North Minch and Fladen ground) and west coast of Ireland (Aran grounds) suggested that *Nephrops* densities have a positive relationship with mud (silt plus clay) sediments. The relationship may have an inflexion when sediments have about 60% of silt plus clay content (also described as a dome-shaped relationship; [48]). This type of relationship was linked to the burrowing behaviour of *Nephrops*, whereby areas with coarse or sandy sediments and low proportions of silt plus clay are usually associated with low population densities, due to coarse sediments being unsuitable for burrow construction. Densities peak in areas with moderately high contents of mud but in very high mud contents, densities may not rise as populations become limited by competition. When overcompensation occurs (decline in population sizes at very high mud contents), this may reflect negative density-dependent effects, i.e. high densities and very fine sediments create sub-optimum conditions. Results for the Aran ground and Celtic Sea are consistent with a carrying capacity being reached. In the Irish Sea, the shape of the response was more dome-shaped than the Celtic Sea and Aran ground, suggesting a stronger density-dependent overcompensation. This also reflects the higher densities observed in the Irish Sea (average value of 1.00 individual m^-2^) compared with other regions (Celtic Sea and Aran ground respectively 0.54 and 0.66 m^-2^; Table 2).

Although the areas compared have generally similar values of predictor variables, the fitted models for individual predictors showed some variations across areas. The may reflect differences between areas in animal behaviour, stock structure or density (see previous paragraph). However we do not rule out the possibility that geographical variations in *Nephrops* burrow densities may also be explained by the relationship of benthic proxies to other factors such as temperature and food availability [49,50] or larval retention and recruitment [51].

Seafloor morphology was also found to be a predictor of *Nephrops* habitat selection, with rugosity and slope being important in four of the five areas, although not the same areas (Table 3). In particular a negative curvilinear relationship was found between *Nephrops* densities and rugosity in the Irish Sea and Fladen ground while a linear trend was found in the Scottish Inshore model (Table 3; Fig. 4). These results suggest that *Nephrops* prefer areas with minimum or gentle terrain variation, which tend to be associated with soft sediments. This confirms previous findings of habitat suitability using Ecological Niche Factor Analysis (ENFA) in the Aran ground, which found that *Nephrops’* best habitat occurred to the foot of areas of steeper slope [52]. Other studies have highlighted the importance of seafloor characteristics for lobster species (*Homarus gammarus*) which suggests that these environmental factors are amongst the most important explanatory variables for crustacean habitat selection [53].

### Spatial Transferability of habitat models amongst areas

Many of the models were able to predict *Nephrops* burrows density in new locations, with the Aran ground, Irish Sea and Celtic Sea models successfully transferring to other areas (Table 5). In general, habitat models can lose some accuracy and calibration when transferred to other geographical regions [17,22]. This can be explained by the fact that a model fitted to data of a specific area (training data) may encounter different conditions when transferred to a new area [12]. Our results demonstrated this but they also suggested cases where it was possible to utilise a habitat model developed in one area to predict *Nephrops* distribution patterns in other areas (Table 5). In some cases, the transferred model predicted distribution just as well as the original (e.g. compare r_s_ 0.73 for the original Celtic Sea model with r_s_ 0.75 for the Aran Ground model applied to the Celtic Sea).

Our results suggest that spatial transferability is relatively decoupled from model ‘fitting’ performance (Tables 5 and 6). This suggests that all habitat models contained a similar amount of predictive information, with the implication that simpler, regression or GAM-based models can perform as well as more complex models and represent a better option for projecting species distributions in new areas. This is in agreement with studies [54] which showed that simpler models (e.g. GLMs, GAMs) dealt better with overfitting than machine-learning models (e.g. Random Forest) and therefore can be more transferable. In addition simpler models may be more effective in conservation planning as they can be easier to carry out or explain to decision-makers.

It is difficult to explain why some models could not be spatially transferred (Scotland Inshore and Fladen ground) while other model transfers seemed insensitive to even the choice between competing models (Table 6). The negative relationships between transferred model and observations for the Scottish areas may result from the different depth and sediment predictive relationships, although it is not clear why these predictive relationships might differ. It may be that the pooling of areas or greater spatial extent of the Scotland inshore area confuses the outcome due to different drivers of *Nephrops* densities within areas. There may also be issues with the accuracy and resolution of the predicted EUNIS habitat maps. A lack of transferability may be used to construct additional hypotheses about the processes causing spatial variation in the study species, feeding into monitoring and conservation efforts.

### Implication of the results for fisheries management

An ecosystem approach and spatial planning have been proposed as tools to sustainably use marine ecosystems and rebuild fisheries worldwide. In order to achieve these goals it is important to improve knowledge on the spatial distribution of commercial fish and invertebrate species and their relationships with the marine environment. In Europe, two key policy initiatives have been put into place to fulfil these objectives: the reformed Common Fisheries Policy (2013) and Marine Strategy Framework Directive [35,36]. Under these frameworks, all Member States will take measures to maintain or achieve good marine environmental status and recovery of fish stocks by 2020. Incorporating knowledge about species distribution drivers into these measures can inform current and future monitoring and conservation programs. The approach presented in the present study, with predictive habitat maps for *Nephrops*, can inform future spatial conservation plans and ICES fisheries advice for an improved long-term regional management of *Nephrops* stocks in the northeast Atlantic. The application of approaches such as the transferability of habitat suitability models can increase our understanding of the main drivers of *Nephrops* habitat selection. Our results highlight the importance of testing for spatial transferability among more than two regions as a model might transfer well to one area but not to others. In order to achieve an effective sustainable management of marine resources, transferability information should be incorporated into the evaluation of species distribution models.

### Conclusions and Recommendations

Under the Marine Strategy Framework Directive (EU, 2010), the assessment of species distribution patterns and habitat utilisation are required to implement lasting and effective conservation and fishery management plans. Identifying species’ habitats is therefore one of the main applications of habitat suitability models for conservation purposes, as has been effectively applied in some cases (see [55]).

Habitat models developed in this study highlighted some of the key distribution areas and habitat associations of *Nephrops norvegicus* in the Northeast Atlantic while testing for the transferability assumption of the models. Environmental factors, in particular depth and sediments, were the important drivers for *Nephrops* habitat suitability in all five study areas. When tested for spatial transferability, most of the models were able to predict *Nephrops* burrows density in new locations but some models could not be transferred at all. This may be related to the data used for model construction, and future developments of this type of approach should investigate if different proxy data or scale issues have an effect on the transferability of habitat models.

Our results show that spatial transferability of habitat models is relatively decoupled from model ‘fitting’ performance and suggests that there are limits to the information in predictor variables. Relatively simple models were as transferable as more complex models, as these predicted species distribution in new areas with similar performances. Therefore striving for a better model fit in this context was counterproductive. Although a number of studies have already examined the transferability of habitat models between different areas this approach is still under development and scarcely applied in marine ecosystems (Table 1). It is clear that further investigations at multiple regional scales are required to understand how spatial transferability of habitat models can be improved.

The current study reflects the objectives of the European Commission (EC) within the framework of an ecosystem-based approach for fisheries management, which aims to identify priority conservation areas to maintain sustainable marine living resources. The habitat models represent a first attempt to predict *Nephrops* densities in several important fishery areas of the Northeast Atlantic and suggest that species distribution mapping and spatial transferability of habitat models can be used to inform future fisheries management plans.
